# “The hardest job you will ever love”: Nurse recruitment, retention, and turnover in the Nurse-Family Partnership program in British Columbia, Canada

**DOI:** 10.1371/journal.pone.0237028

**Published:** 2020-09-08

**Authors:** Karen A. Campbell, Natasha Van Borek, Lenora Marcellus, Christine Kurtz Landy, Susan M. Jack, on behalf of the British Columbia Healthy Connections Project Process Evaluation Research Team

**Affiliations:** 1 School of Nursing, McMaster University, Hamilton, Ontario, Canada; 2 School of Nursing, University of Victoria, Victoria, British Columbia, Canada; 3 School of Nursing, York University, Toronto, Ontario, Canada; University of Auckland, NEW ZEALAND

## Abstract

**Background:**

Nurse turnover is a significant issue and complex challenge for all healthcare sectors and is exacerbated by a global nursing shortage. Nurse-Family Partnership is a community health program for first-time pregnant and parenting girls and young women living in situations of social and economic disadvantage. In Canada, this program is delivered exclusively by public health nurses and only within a research context. The aim of this article is to explore and describe factors that contribute to recruitment, retention, and turnover of public health nurses delivering Nurse-Family Partnership in British Columbia, Canada between 2013 and 2018.

**Methods:**

Interpretive description was used to guide sampling, data collection and analytic decisions in this qualitative component drawn from the British Columbia Healthy Connections Project mixed methods process evaluation. Semi-structured, individual interviews were conducted with 28 public health nurses who practiced in and then exited Nurse-Family Partnership.

**Results:**

Nurses were motivated to join this program because they wanted to deliver an evidence-based program for vulnerable young mothers that fit with their personal and professional philosophies and offered nurse autonomy. Access to program resources attracted nursing staff, while delivering a program that prioritizes maintaining relationships and emphasizes client successes was a positive work experience. Opportunities for ongoing professional development/ education, strong team connections, and working at full-scope of nursing practice were significant reasons for nurses to remain in Nurse-Family Partnership. Personal circumstances (retirement, family/health needs, relocation, career advancement) were the most frequently cited reasons leading to turnover. Other factors included: involuntary reasons, organizational and program factors, and geographical factors.

**Conclusions:**

Public health organizations that deliver Nurse-Family Partnership may find aspects of job embeddedness theory useful for developing strategies for supporting recruitment and retention and reducing nurse turnover. Hiring nurses who are the right fit for this type of program may be a useful approach to increasing nurse retention. Fostering a culture of connectivity through team development along with supportive and communicative supervision are important factors associated with retention and may decrease turnover. Many involuntary/external factors were specific to being in a study environment. Program, organizational, and geographical factors affecting nurse turnover are modifiable.

## Background

Nurse turnover is a significant issue and complex challenge for all healthcare sectors and is exacerbated by a global nursing shortage [1–3]. In Canada, nurses comprise one third of the health care workforce and are the largest body of regulated health professionals [4,5]. Yet the Canadian Nurses Association suggests that healthcare needs are not being met by the current workforce and new policies need to be implemented to address the anticipated shortage of almost 60,000 registered nurses by 2022 [6]. Furthermore, the Canadian Institute for Health Information reports that the annual growth rate of employed regulated nurses in 2017 was the lowest in the previous decade [7]. Given the existing and increasing nursing shortage, policy makers and managers will need to address issues associated with retention and recruitment of registered nurses.

Solutions to the nursing shortage include improving recruitment, reducing turnover and retaining nurses [6]. It is estimated that nurse turnover costs Canadian employers 1.2–1.3 times the annual salaries of registered nurses [8]. Beyond the cost and negative effects on budgets, nurse turnover is also a significant problem for human resources planning [7]. While there is extensive research on nurse retention, most focuses on hospital settings [9,10]. With only three percent of the 300,000 Canadian registered nurses employed in community-based public health positions, there has been a lack of corresponding attention to public health nursing retention research [7]. The need to recruit and retain nurses is also of considerable importance to this sector of the health system [11]. The aim of this study was to explore and describe nurses’ perceptions about factors that contribute to the recruitment, retention, and turnover of public health nurses delivering a nurse-home visitation program, Nurse-Family Partnership^®^ (NFP), in British Columbia, Canada.

### Nurse turnover and retention

The definitions of nurse turnover and retention are inconsistent in research and reviews; turnover and attrition are often used interchangeably, making comparisons difficult [9,12]. For the purposes of our analysis, we have operationally defined *turnover* as nurses voluntarily or involuntarily leaving their position [3,8,12]. We acknowledge that turnover occurs for voluntary reasons, such as personal or professional decisions to leave or change a position, but also for involuntary reasons, including program closures, retirements, or termination of employment [12]. Conversely, the focus of nurse *retention* is to prevent nurse turnover and is defined as the intent to continue employment in a nurse’s current role [10,13]. In many research articles, including ours, these concepts are studied together.

Retention and turnover of nurses is a complex human resource phenomenon influenced by multiple intersecting individual and contextual factors. An individual’s sense of job satisfaction has been identified as a key factor in nurse retention and turnover [1,14]. Studies have also suggested that personal (personality, burnout), job (perceived empowerment, career advancement, remuneration), and organizational characteristics (management style, cultures of stress) are associated with nurse turnover [1,14–17]. A recent meta-analysis identified that a lack of supportive and communicative leadership, strong workplace connections, and organizational commitment are the strongest predictors of voluntary turnover [17]. These findings are reinforced by similar conclusions from studies of nurse retention, and many systematic reviews concluded that supportive relationships with co-workers and leadership, pay parity, and quality workplace environments are the most effective strategies and contextual conditions favourable to retaining nurses [9,18–23].

Although there is ample literature related to the human resources issues of turnover and retention in acute and long-term care health settings, little is known about these issues within community-based public health departments, including those that offer nurse home visitation programs [10,15]. Given increased demand for such nurses in the current and planned shifts to population health and primary health care and the ongoing context of nursing shortages, retention and turnover issues will become increasingly challenging for leadership and public health managers [11]. Public health nurses in Canada are baccalaureate degree prepared and vital in promoting, protecting, and preserving health for individuals, families, communities, and populations [2,24,25]. Issues of social justice, client advocacy, and health equity are paramount in public health nursing and require a high level of nursing skill and knowledge [24]. Yet public health nursing is not typical of traditional point of care nursing, adding to recruitment difficulties among new nurse graduates who have had limited exposure to community care environments during their undergraduate nursing education programs [26,27]. Jones and Gates [13] have called for more research on turnover and retention from different areas of nursing, such as public health, to highlight and minimize the costs and benefits of turnover.

Of the few studies focused specifically on public health nursing, many are from an American perspective. Yeager and Wisniewski [11] conducted a cross-sectional study in the United States (US) to examine nurses’ decisions to work in public health. They found that nurses were more likely to remain in public health positions when their values aligned with the organization’s mission, they were able to be innovative in a flexible environment and had access to continuing education opportunities. Findings from two studies conducted by Leider and colleagues [28,29] suggest that American public health workers enjoyed their roles but did not feel adequately compensated. Despite the differences between Canadian and American health systems, wages were also found to be an issue for public health nurses in two Canadian studies [10,30]. If new graduates are to fill employment demands, agencies must address the loss of institutional knowledge as turnover occurs and identify a means to retain new public health practitioners [28].

Existing Canadian studies found that public health nurses were most satisfied when they provided direct client care, received positive client feedback, and when they perceived that their work made a difference [30,31]. Henderson, Betkus, and MacLeod [10] surveyed public health nurses in British Columbia and learned that intent to leave was associated with factors of remuneration, retirement, and family needs more than job satisfaction. However, this cohort included only nurses delivering services in rural and small towns; a sector of the workforce known to have unique experiences specific to their geographical context [32]. In 2005, a comprehensive examination of Canadian community-based nurses uncovered that all sectors of community nursing (i.e., home care, public health, and community care access centres) were concerned with inadequate staffing, client complexity, providing nursing care to vulnerable families with multiple health issues, and “difficult clients” [25, p. 184]. Issues specific to public health nurses affecting retention included constant change and uncertainty associated with programs being offered [25]. However, it is unknown whether these concerns reflect nurses’ experiences in the current climate of Canadian public health nursing.

NFP is a community health program for first-time pregnant and parenting adolescent girls and young women coping with socio-economic disadvantage [33]. Within the Canadian context, since 2008, the program has been delivered within multiple research contexts in order to adapt then evaluate the program in this context [34,35]. Baccalaureate-prepared public health nurses regularly visit families starting early in pregnancy (<28 weeks gestation) and with visits continuing until the child’s second birthday [33,36]. Canadian public health nurses have historically been trusted to care for mothers and their infants [37,38] and have a range of knowledge and skills to address the complex health and social needs of families experiencing multiple challenges [24,37]. NFP supports building strong therapeutic relationships between nurses and clients to improve pregnancy outcomes, reduce child maltreatment, improve child mental health and development, and improve mothers’ life circumstances [33,36]. In British Columbia, with support from the Ministry of Health, through the British Columbia Healthy Connections Project, regional health authorities are implementing and delivering NFP with fidelity to the program’s core model elements [36]—as part of the first randomized controlled trial evaluating NFP’s effectiveness in Canada [35].

NFP has been extensively evaluated in the US and has demonstrated effectiveness in positively influencing a significant number of reproductive, child and maternal health outcomes [39]. In addition to establishing the evidentiary foundations to support the efficacy of this program in improving health outcomes among young first-time mothers and their children, research has also been conducted to develop and evaluate innovations to improve program delivery. Significant attention has been paid to understanding and then addressing issues that influence client retention in the program [40,41]. Through this work, it has been established that client retention in the NFP program is optimized by ensuring one consistent nurse home visitor throughout the length of the program; as a consequence nurse turnover is strongly associated with client attrition or lack of engagement in the program [42–45]. Therefore, nurse retention is vital to NFP success.

Our understanding of the contextual and program factors that influence NFP nurse home visitor retention and turnover is limited to a few studies conducted in the US and Canada. Zeanah and colleagues [45] conducted focus groups to study NFP nurses’ provision of mental health care to clients in Louisiana, US. They noted that retaining experienced NFP nurses who were able to form and maintain client relationships was crucial to program impact. They suggested that a loss of nurses from the program may increase organizational costs related to the subsequent training of new staff. Zeanah et al. recommended finding opportunities for nurses to formally and informally process the emotional work of NFP to potentially help with nurse retention [45]. In another qualitative study, Lewis [46] found that nurses were drawn to the NFP program because of a desire to work with pregnant/parenting young women and their children who were experiencing socio-economic disadvantage—as well as having greater role autonomy, job flexibility, and opportunities to develop relationships with clients [45,46]. The emotional toll, high caseloads, insufficient resources, and inadequate salaries were some of the challenges associated with NFP nursing in these two studies from the US [45,46]. Similarly, in Canada, a qualitative secondary analysis of data from an NFP pilot study in Ontario, augmented by additional interviews with nurses, found that workload and workplace factors increased their stress [47]. However, nurses were highly satisfied by their ability to develop strong client relationships and observe their clients’ successes [47]. This study identified program structure (including a model of reflective supervision), a shift in nursing philosophy, and the support of NFP colleagues as factors supporting nurse retention. Understanding the emotional labour involved in delivering NFP, Dmytryshyn and colleagues [47] recognized that turnover can be associated with burnout, fatigue, and vicarious trauma and acknowledged the need for strategies to better support nurses.

Currently, we lack an in-depth understanding of the organizational, team, and individual factors that influence the capacity of the NFP program to recruit, retain, and reduce the turnover of public health nurses employed within this targeted nursing intervention. Identification and exploration of these factors is a first and essential step in the process of developing human resource strategies to ensure that implementing public health agencies maintain their capacity to successfully deliver NFP. This analysis draws from a sub-set of qualitative data from the larger mixed-methods process evaluation conducted in the context of an ongoing trial to examine effectiveness. The focus of this analysis is to identify and describe NFP public health nurses’ perceptions of contextual, organizational and individual factors that influence the workforce cycle. We also documented their recommendations for how NFP implementing agencies can focus their efforts on nurse recruitment, retention and limiting turnover.

### Evaluation of the NFP in British Columbia, Canada

Evidence of NFP effectiveness in Canada will support national extension of this service and therefore the employment of more public health nurses. Understanding the factors influencing nurse recruitment, retention, and turnover in this program is vital to sustainable establishment of NFP in Canada. In Canada, NFP was adapted and piloted for acceptability and feasibility between 2008–2012 in Hamilton, Ontario [34]. While NFP has been extensively evaluated in the US and has demonstrated effectiveness in positively influencing a significant number of reproductive, child and maternal health outcomes [39], the effectiveness of this early intervention program is unknown within the context of Canada’s universal healthcare system. As such, the British Columbia Healthy Connections project (BCHCP) was launched in 2012 and comprises a randomized controlled trial (RCT) [35] evaluating NFP’s effectiveness in a sample of 739 families from four participating regional health authorities. The BCHCP also involves an adjunctive process evaluation to document how NFP is implemented and delivered by five unique health authorities [36]. This paper reports on findings drawn from data collected from public health nurses who participated in the process evaluation. The specific research questions addressed by this analysis are:

1. What factors influence decisions to apply to, remain in, and leave NFP roles for public health nurses who exited the NFP program?
2. What recommendations do public health nurses have related to developing and sustaining a high quality NFP public health nurse workforce?

## Methods

### Study context

Public health services in British Columbia are delivered through five regional health authorities and are guided by mandates from the provincial Ministry of Health [48,49]. Each of these health authorities participated in the process evaluation and assigned PHNs and supervisors to complete the NFP education program and deliver the program to girls and young women who met the RCT eligibility criteria [35,36].

### Design

Qualitative data were drawn from the BCHCP mixed methods process evaluation [36]. An interpretive descriptive approach was employed to guide all methodological decisions related to data collection and analysis because of its utility and practicality in qualitative health research [50,51]. Interpretive description draws on the disciplinary knowledge of the researchers to generate research findings that are clinically relevant and meaningful to applied practice [50,52]. The core research team for this analysis included four registered nurses with significant expertise related to public health nursing and home visitation programs including NFP (KC, LM, CKL, SJ) and a research coordinator with a graduate degree in public health (NV). The tenets of interpretive description do not restrict researchers to ill-suited theoretical frameworks but encourage the use of methods or theories that support the development of practical and applicable research outcomes [50,53].

### Participants

The entire population of public health nurses delivering NFP in British Columbia were invited to participate in the process evaluation. Study eligibility criteria included: 1) completed or completing the NFP education; 2) delivering the NFP intervention to participants in the BCHCP; and 3) English-speaking [36]. From this population of nurses, the sub-sample for this analysis consists of public health nurses who subsequently left their NFP positions during the period of February 2015-May 2018. Changes in a nurse’s status was communicated by the NFP supervisor to the research coordinator for the BCHCP process evaluation, who then contacted and invited the nurse to participate in an exit interview prior to their last day in the role.

### Data collection

The purpose of the exit interview was to explore nurses’ experiences and perceptions of their position in the NFP program, including motivation for applying to NFP and reasons for leaving, as well as to gather their recommendations for strategies to promote nurse retention within the program. Participants were interviewed via telephone at a private space in their workplace by the research coordinator. Telephone interviews can create challenges in qualitative research, particularly through the loss of nonverbal cues (e.g. anxious behaviours), inability to contextualize data (e.g. environment or participant characteristics), and difficulties building rapport [54]. To mitigate these potential risks, two authors (NV, SJ) visited work sites and had previously interviewed study participants, thus developing interviewer-participant relationships. Despite potential limitations, some research suggests that participants may have enhanced comfort via phone [54]. In addition, telephone interviews were cost-effective and convenient to rapidly schedule for participants who were leaving their positions. The interview guide was developed and edited by members of the BCHCP Process Evaluation team, which included an International NFP Consultant who provided content expertise. The guides were reviewed regularly for any potential changes (see S1 File). Interviews ranged in length from 33 to 85 minutes, were digitally recorded, and transcribed verbatim with all identifying information removed. Participants completed a short demographic form at the time of the interview.

### Analysis

Using the principles of interpretive description [50], initial analyses were conducted by two members of the research team (NV, LM) which included multiple readings of the transcripts and discussions between the researchers to make meaning of the data. The coding process began inductively through a process of open coding and categorizing. A codebook was developed and used to compare coding decisions and facilitate dialogue about emerging patterns. Individual codes were then collapsed into higher level categories to identify the main themes across all interviews. Each transcript was then revisited and narratives of experiences relating to recruitment, retention and attrition were extracted. Other analytical strategies used to bring meaning and abstraction to the data included journaling, building matrices, and diagramming.

Key narratives and quotations were extracted from the transcribed interviews and reviewed by all authors for consistency and consensus in analytical meaning. All members of the research team have significant experience in qualitative research and were involved in determining relationships and patterns in the data used to conceptualize the findings in a way that is meaningful for public health nurses and illuminates the factors associated with NFP nurse turnover, recruitment, and retention issues. Findings were substantiated through the thoughtful clinician test, which included a variety of public health nurses and other individuals familiar with NFP reviewing outcomes and recommendations for clinical relevance [50].

### Ethics

The BCHCP mixed methods process evaluation received research ethics board approvals from ten institutions: the five participating health authorities, four universities where BCHCP researchers are affiliated, and the Public Health Agency of Canada. All study participants provided written and verbal consent prior to the interview and were informed that their participation in the study was voluntary. Public health nurses did not receive compensation for their participation in the study and completed the interview during assigned work hours.

## Results

Overall, a total of 82 public health nurses participated in the BCHCP process evaluation (2013–2018). Between February 2015 and May 2018, when data for this analysis were collected, 38 nurses across 5 health authorities left their NFP positions and exit interviews were conducted with 28 of these nurses (73.6%). Of the 10 nurses not participating in an exit interview reasons included: unable to set up interview before NFP exit date (n = 9); and, nurse did not respond to invitation (n = 1). Participant characteristics are summarized in Table 1.

**Table 1:** Public health nurse characteristics.

Characteristics	% (n)
Highest level of education	
Baccalaureate degree	89.3% (25)
Master’s degree	10.7% (3)
Average nursing experience (in years)	14 (range 2–40)
Average home visiting experience (in years)	8 (range 0–27)
Average public health experience (in years)	9 (range 0.5–28)

Through the accounts of 28 public health nurses, we observed that these nurses were enthusiastic about the opportunity to deliver NFP. However, despite their satisfaction with NFP, multiple voluntary and involuntary/external factors challenged their capacity to remain in the program. An in-depth examination and analysis revealed three key stages that emphasize the workforce cycle for NFP public health nurses: joining the NFP team, developing relationships and new skills and deciding to leave. Stages represent the processes of recruitment, retention, and turnover and key findings are summarized and presented in Table 2.

**Table 2:** Key factors for NFP nurse recruitment, retention, and turnover.

Recruitment: Joining the NFP Team
• Fulfills professional goal of working with populations experiencing vulnerability which provides professional fulfillment • Creates an opportunity to work within, and deliver an evidence-based nursing program • Provides opportunities to develop and refine nursing knowledge and skills through completion of NFP core education • Have access to large number of program resources (visit-to-visit guidelines, facilitators) to use to tailor home visit content to meet identified family needs • Support for nurse autonomy with respect to planning and delivering care for families and to practice self-care • Fits with personal and professional philosophies of nursing care
**Retention: Developing Relationships**
• Program structure and model elements emphasize and prioritize the importance of developing and maintaining relationships with families • Delivery of an intensive, client-focused home visitation program through regular home visits allows nurses to witness the attainment of positive health outcomes among highly vulnerable families • Being part of a strong, cohesive team of nurses which allows for regular debriefing, consulting about challenging clinical situations, and receipt of support from individuals who “understand” program demands
**Retention: Developing New Skills**
• NFP program model and focus on multifaceted aspects of promoting maternal, child and family health creates a context where public health nurses are working at the full scope of practice • Opportunities for ongoing- and regular professional development to address nurse-identified educational needs • Engaging in regular reflective supervision can provide nurses with a supportive environment to practice reflection and evaluate nursing care
**Turnover: Deciding to Leave**
• Experiencing symptoms of compassion fatigue, burnout, vicarious trauma, or moral distress • Difficulty balancing competing priorities within limited time allowances (i.e. for documentation or travel) • Personal reasons (e.g. moving from service delivery area, retirement) • Organizational factors (e.g. changes in program or staff movement, unhealthy work environments)

### Recruitment: Joining the NFP team

Participants emphasized that the opportunity to deliver an “evidence-based program” and potentially have a long-term impact on families’ lives motivated them to join the NFP team. Throughout the interviews, nurses referred to NFP as an “evidence-based program,” even though the effectiveness of the program in British Columbia has not yet been established. An experienced, end-of-career nurse described what motivated her in this role,

I really felt like I wanted to do something in my career that I felt would really be able to make a difference and I felt a real connection with just core values and then the NFP client-centered principles, and just the social impact that NFP had not only for the client's family but even then through generations.

This perception of NFP being a program that “makes a difference” for families is underpinned in the NFP core education where NFP nurses in British Columbia are introduced to the positive outcomes measured in the US trials. Nurses expressed fulfillment in working in the area of maternal-child health and with high-priority populations, such as adolescent girls and young women experiencing complex, chronic health and social issues. A nurse explained the connection she had to the clientele in NFP as a draw to this type of work,

I love young moms and the connection I was making with some of the vulnerable ones … Knowing that that was basically my clientele was a huge draw for me. Providing evidence-based nursing care to young mothers was a motivating factor for nurses who applied to work in NFP.

Previous experience working in public health parenting programs without adequate time, clear guidelines, and resources to address complex client needs piqued nurses’ interest to apply to work in the NFP program. An experienced public health nurse shared her discontentment with prior practices,

One [reason for applying to NFP] was maybe the dissatisfaction of my ability to work with vulnerable populations in general public health. I just found that it was getting more challenging to find the time to be available to the families that needed us most because we were spread so thin with other duties. And also, there was no template for which to work with these families, so everybody did their own thing and I just felt that there was a better way but just didn't know what that better way was.

In contrast to having limited guidelines to structure their home visiting practices in general provincial home visiting programs, some nurses expressed frustration about previous experiences that did not allow for them to consistently apply their nursing skills, judgement, or work at their full scope of practice. The lack of autonomy within their previous nursing practice and its increase in scripted responses and “checklist nursing” was described in this quotation:

I had done the other jobs in public health for a long time and it was getting more narrow and narrow all the time. We were told what to do and when to do it. A lot of it was like phones and scripts. So, it wasn't a very interesting job.

Participants hoped NFP would provide them with the opportunity to complete a robust program of nurse education, improve their knowledge and skills, and have access to visit-to-visit guidelines, tools, and resources to integrate into home visits.

Nurses found the potential to become home visiting experts and experience a sense of personal and professional fulfillment in providing a program informed by theory, based in evidence, and guided by focused client-centred principles appealing. Furthermore, they valued the education and support offered by the NFP program model. Participants, even those with significant maternal-child expertise, viewed NFP as an opportunity to increase their skill set working with high priority populations, “I was very interested in the program because it was an opportunity to learn new skills and to become a bit of an expert in this area.” Nurses appreciated the advanced education they received. Even more significant, nurses highly valued that the program model allowed for, even encouraged and provided support, time, and flexibility, for nurses to do whatever was required to establish, build and maintain a strong, consistent therapeutic relationship with the client. Nurses new to public health practice were also drawn to NFP for similar reasons:

There's a lot of support and backing from supervisors and management to get education to broaden ourselves, and also to do self-care. You don't see any of those things in the hospital really. So that was what I was lacking and what I needed and so it totally fit the need for me in NFP.

Nurses exiting the program provided insights into recruitment strategies and stressed the importance of job fit. They perceived that the personal qualities necessary to be successful in the NFP role included being flexible with how they implemented program elements and exhibiting openness and self-awareness. The necessity of job fit was explained:

If [a nurse] doesn’t fundamentally have the right attitude—if they're not open, if they're not able to roll with the punches, if they're not able to really critically look at themselves, their boundaries, their reaction to things, if they don't have that insight or aren't willing to develop it, they won't get, or last in, the program.

Additionally, there was a set of past professional nursing experiences that participants identified as critical considerations when NFP teams or nurse supervisors were advertising and recruiting for new nurses to join the team, including: 1) practice with populations experiencing adversity or marginalization; 2) confidence and competence in home visiting; 3) demonstrated abilities to establish professional boundaries with clients; 4) understanding of system level facilitators and barriers to meeting multiple client health and social needs; and 5) the ability to frame and deliver care and services from a strengths-based, rather than a deficit-focused, approach.

### Retention: Developing relationships

The nurses were all asked to reflect on their time delivering NFP to pregnant/parenting girls and young women and their experiences working with other NFP team members, and then to identify the key factors that they perceived, to influence nurses in general, to want to remain working in the program. There was broad consensus that the two primary reasons public health nurses are motivated to continue to work in this program are the opportunities to develop genuine therapeutic relationships with families as well as to work at their full scope of nursing practice. As one nurse concluded: “I loved the fact that it was a long-term relationship that you’d be building and that there were so many educational opportunities that came with NFP.” Within these two conditions, nurses expressed a deep commitment to making a difference in the lives of their clients and were encouraged by the opportunity to have a greater impact through their work in the NFP program.

Nurses identified that the nature and depth of the nurse-client relationship established within the context of this program contributed to their overall job satisfaction. NFP nurses valued building trusting relationships with clients and recognized the importance of establishing foundations for a therapeutic relationship: “I'd say the relationships and getting close to people that are usually very guarded and don't trust a lot of people. That they trust you and then they work with you.” In addition to relationship building, nurses were highly satisfied when they were able to witness positive client outcomes: “When you have success with your clients, it’s brilliant… When you have those moments where your clients do something amazing and maybe I had a part in that. Look at this—I’ve made a difference!” Observing mothers becoming more confident in their parenting skills, infants meeting their developmental milestones, and families breaking inter-generational cycles of poverty or trauma were valued by nurses. These factors were identified by nurses as retention factors (for the time they remained in the program).

Participants also pointed out the importance of contextualizing the concept of success in the NFP program. One nurse shared how NFP changed her nursing practice: “It’s liberating as a nurse to be able to do that [strength-based work] because [in comparison to other programs] … I always felt the emphasis as a nurse [was] you're always looking for problems.” At the individual client level, participants reported successes including positive parent/child attachments, leaving abusive partners/negative influences/toxic relationships, quitting or reducing their use of substances (i.e. alcohol, tobacco), attending/completing school, acquiring employment, and receiving mental health support. For one public health nurse, the connection that she had to her client and the ability to focus on, and celebrate, her strengths were evident:

Just watching my girls succeed and it looked different for each one of them. I mean one of my girls, the baby was apprehended multiple times and I think permanently after the [NFP] program, but her baby was born with an intact brain and that's something she [the client] wasn't given. She avoided drugs and alcohol throughout her entire pregnancy. And her baby got a normal brain, and this is a mom that was born with FAS [fetal alcohol syndrome]. So even though she didn't successfully parent maybe in the way we look at, I saw her breaking the cycle and it was so neat to see those little changes and how that will impact not only her daughter but her daughter's children too.

The repetition of the term “my girls” in this participant narrative reflects the commitment and connection that the nurse felt towards her clients. Despite a child apprehension, the nurse was able to identify client strengths that she attributed to NFP exposure. Client success in NFP was not an all or nothing binary outcome. Because NFP is client-focused and strength-based, public health nurses could celebrate all successes.

Positive working relationships with the NFP team and supervisor were generally experienced as supportive and encouraged participants to remain in their position. Required weekly reflective supervision was consistently mentioned as a benefit to being in NFP: “Some of the things that are very helpful to retaining nurses, I think, are supportive supervision and regular reflective [supervision].” Engagement in reflective supervision provided dedicated time to critically reflect on situations and receive essential support from supervisors to advance nursing practice. One nurse shared why reflective supervision and a supportive environment was so important: “NFP is a program where, as the practitioner, you need a ton of support because it's heavy work.” The notion of team cohesion and positive working relationships as a means to deal with the challenging work of NFP was echoed: “I think the team meetings and the reflective practice are huge for retention and, well, decreasing burnout. But also, for growth as well, the case conferences that we do are really engaging, and I actually miss them.” Regularly reflecting, discussing, and debriefing with supportive colleagues and an assigned NFP supervisor who understand the nature and stresses of the program allowed nurses to feel connected and supported in their work.

### Retention: Developing new skills

Participants recognized that professional development, such as the completion of NFP core education and the availability of ongoing learning opportunities, supported them to advance their professional nursing knowledge and skills. Nurses attributed their clinical successes to the intensive educational opportunities provided and suggested that it may be a factor for ongoing retention of NFP nurses: “I really enjoyed the ongoing learning and education that was part of the NFP; that was a real retention piece for me.” Opportunities to gain and apply knowledge were found to enhance professional growth for public health nurses: “Using that research base and developing knowledge and skills more intensely in a way of applying theory and research … to develop as a professional.” Being in a role that supported professional growth and development was important to nurses.

With advanced knowledge and in a supportive environment, nurses were able to work at their full scope of nursing practice. This milieu allowed for regular, in-depth nursing assessment, planning, intervention, and evaluation: “I felt like I was using all my nursing muscles instead of just a few of them.” The ability to learn new assessment techniques used in NFP and apply them in their public health nursing practice was professionally stimulating. Another nurse reflected on her time in NFP: “It's been a really interesting role that I will definitely take a lot from.” Many nurses noted that NFP was the most challenging nursing position they had experienced: “[During training] I was like, ‘oh it can't be that hard’. It can't be any harder than what I've already had to deal with as a nurse. And, no … I've lived that.” Providing complex nursing care to NFP clients using an array of new resources, skills, and knowledge was meaningful to nurses.

### Turnover: Deciding to leave

Participants identified a variety of voluntary and involuntary/external factors (Table 3) that contributed to their decision to leave the NFP program. Contributing factors are presented and described in this section.

**Table 3:** Circumstances that contributed to PHNs leaving an NFP position.

**Involuntary/External Decision to Leave NFP Team**	
Organizational	Health authority restructuring
	Reallocation of nurse resources to another health unit program
	Position terminated with no known rationale
	Union grievance resulting in staff movement
**Voluntary Decision to Leave NFP Team**	
Organizational	Lack of job security
	Filling a temporary position
	Uncertainty regarding sustainability of NFP position post-BCHCP RCT
	Misunderstanding of RCT study timeline
	Negative work environment
Personal	Retirement
	Family needs
	Health issue
	Moving out of health authority area
	Another employment opportunity
Program	Perceived lack of adequate supervisory support
	Lack of time to meet program demands (e.g. increased caseload, travel time, preparation for visits, locating clients, documentation)
	Lack of fit between PHN preferred assignment and demands of NFP program (e.g., working exclusively with families with multiple complex needs)
	Caseload management (particularly when assigned only part-time to NFP)
	Experienced isolation due to geographical factors
	Too much driving/covering broad geographic area
Unknown	No reason given

*Some participants described more than one reason for leaving their position.

#### Involuntary/external circumstances

Nine out of the 28 nurses interviewed identified at least one involuntary or external factor as the primary reason they were no longer assigned to an NFP role. Involuntary or external reasons for job turnover, as described by the nurses, included: 1) health authority restructuring; 2) reallocation to another program; 3) union grievance; and 4) position terminated for no known reason. For one health authority, the NFP program was discontinued as the health authority restructured programs: “My health authority has decided that, with their switch to a new model of delivering public health services, that [NFP] does not fit with their new model, and so they've deleted my position in the program.” Under these circumstances, some nurses did not want to exit NFP: “I'm not leaving my position, I'm being displaced.” How leadership communicated decisions about who would exit the NFP role mattered to how the nurse experienced it (i.e. collaborative decision making versus no control). Regardless of the reason for the involuntary turnover, supportive communication about program changes and opportunities to process them influenced how changes were received and experienced by participants.

Institutionally, union regulations, system reorganization, and reallocation of nursing resources were structural barriers that imposed constraints and resulted in involuntary turnover. Participants perceived that interference from the nurses’ union may have caused hiring practices that could be responsible for increased turnover. Specifically, some nurses explained their perception that the union mandated that seniority ranked over personal and professional attributes when hiring into NFP positions and existing positions were grieved. A concerned nurse stated: “[It’s] almost like [the union] is treating [hiring] like a nurse is a nurse is a nurse. And I don't believe that's true.” The importance of hiring for job fit during recruitment was reinforced when participants discussed turnover. In one instance, the reason for being asked to leave NFP was unknown to the participant.

#### Organizational factors

In BC, NFP was implemented by four regional health authorities as part of the BCHCP RCT evaluation of the program’s effectiveness; meanwhile (since 2017), four health authorities are continuing to implement NFP as part of enhanced public health services while awaiting RCT final outcomes. At the front-line practice level, however, limited communication about the long-term delivery of NFP within local offices increased frustrations for some NFP teams. While participants recognized that the Ministry of Health and the BCHCP research team may have conveyed information about long-term NFP planning possibilities to health authority leadership, nurses disclosed that they did not consistently receive clear messaging at the frontline. Participants perceived the demands on busy supervisors could have led to a lack of information-sharing and shared how team members responded: “We had this joke … that we were mushrooms; we were kept in the dark.” Participants feared the unknown and were challenged when they were in environments that lacked transparency, particularly when higher-level decisions might have the potential to impact the roles and positions within which they work.

The transition from delivering NFP as part of a research study to direct delivery of NFP as an integrated public health program was also handled differently amongst health authorities, which led to a range of positive and negative participant experiences, as it related to involuntary turnover. For sites where there was a reduction in NFP nurses, when nurses were included in the decisions about who would leave NFP, through respectful dialogue, the experience was considered positive:

We were all approached. It was very respectfully done … [the supervisor] explained what was happening. And so, I thought well for me, getting close to the end of my nursing career, that it would make sense that I would be the one who would step aside.

Communicating program changes and resource allocation, with clarity and transparency, allowed for a more positive experience for exiting NFP nurses.

#### Geographical factors

For participants working in smaller communities, isolation was a factor influencing participants’ decisions to leave. While the majority of NFP nurses reported not being co-located at the same office as their supervisor, a few were also the only NFP nurse located within an office and expressed feelings of isolation. Participants at these lone nurse sites were concerned that they were at a greater risk of burnout if they stayed in their positions. In addition to lacking an NFP-supportive team physically co-located with them, these nurses often lacked adequate coverage during illnesses or vacations. One participant shared the following suggestion:

[Make] sure there's no lone nurses. I mean, I think, that it's really scary. And I actually have said that to my colleague that's taking over. ‘You're going to be really tired you know. There are going to be days where you're going to be like, I don't feel great and normally would've been like stay home but I got to see so and so and I know she's not in a great place and so I got to be on my game’.

The pressure to be present for clients without readily available and in-office NFP team support was identified by nurses as one factor that contributed to turnover for participants working as the sole NFP nurse in their area.

#### Personal factors

Personal reasons were cited at times for choosing to leave the NFP program. These included family needs, retirement, taking another position for career development, and relocating outside of the office catchment area. Other nurses shared that the working environment was stressful or unsupportive and within the context of their own health or a family member’s health, the decision to leave was necessary. Some retiring participants stayed connected to the program through ongoing employment in casual relief positions: “I'm not leaving because I don't like my job, I'm leaving because of my age … But I've agreed to go back as a casual a little bit.” Many of the participants noted that NFP was a good nursing position: “I would say that the program is everything you've ever dreamed of for your nursing practice.” Despite their reasons for leaving, many nurses enjoyed the experience and urged implementing organizations to address issues associated with turnover.

#### Program factors

At the clinical level, the ongoing challenges of engaging “hard-to-reach clients” while managing a complex caseload and dealing with the time constraints associated with traveling to clients’ homes and completing the subsequent documentation influenced nurses’ decisions to leave:

I was done with the role, feeling burned out … expected to do a lot of driving and not being supported with having different worksites. And just feeling unable to properly get my charting done and properly have time to prep for visits.

Many nurses who voluntarily left the program shared concerns about being overburdened by the work of NFP. Where reflective supervision was not being provided adequately for nurses this became a contributing factor for attrition. Supervisor turnover for some nurses also contributed to lack of adequate support. Where supervisors had no prior understanding of NFP, reflective supervision was less than optimal.

In some contexts, during periods of carrying smaller caseloads nurses were returned in a part-time capacity to non-NFP public health nursing. These participants expressed feeling less confident in the multiple roles assigned due to limited exposure, “I felt so scattered. I didn't have my foot in regular public health any more … And maintain the connection with public health and be able to also try and learn the NFP when your brain wasn't functioning anymore.” This was also the experience of some nurses who were regularly working in a dual role of both NFP nurse and generalist public health nurse. During busy public health times, such as school or flu immunizations, NFP nurses were expected to designate specific workdays to each role. NFP nurses who also had other non-NFP assignments experienced frustration and job dissatisfaction:

It was very clearly told to me at the beginning that—when I'm NFP, I'm NFP and when I'm [communicable diseases] I do [communicable diseases] and it does not cross. And, that's a challenge because the realistic part of it is if a family, an NFP family, calls me in crisis—I answer my phone. I can't really say, ‘well I'm sorry but I can't help you with this until Friday. I know it's only Tuesday but you're going to have to just figure it out.’ And, similarly I can't just not answer my phone or respond to [communicable diseases] things … It's kind of you know unethical or a pull between the two jobs.

NFP nurses who had multiple assignments often experienced concomitant negative consequences (i.e. stress, and physical and mental strain), which contributed to their leaving NFP.

The stress associated with supporting young mothers navigating a range of complex crises also created the potential for nurses to experience vicarious trauma. Because of the frequency and intensity of home visits, and the strong relationships formed with clients, nurses found it difficult when clients experienced challenges: “You feel like you're making so much headway with the client and … then they go off the rails. And it's like everything they've been working towards falls apart … and that's pretty hard to witness.” Participants described experiencing a great deal of stress and worry over client and child safety, and feelings of moral distress when clients disclosed information around perceived risks that they had no ability to change. As one nurse characterized her work in the NFP program: “The hardest job you’ll ever love … really tough work emotionally.”

## Discussion

Our findings highlight the factors associated with recruitment, retention, and turnover from the perspectives of Canadian public health nurses who were employed in, and then left, the NFP program. Furthermore, we build on and extend previous research on the experiences of NFP nurses and influences on intent to remain in or leave the NFP program [45–47]. As part of a process to adapt, pilot and evaluate the NFP intervention in British Columbia, Canada, health authorities are investing significant resources to educate and support public health nurses to deliver this public health program. While comparable Canadian figures are not available, in the US the first-year education costs alone are estimated at over $5000 for each NFP nurse and over $6000 for supervisors (USD) [55]. Therefore, it is important for implementing organizations to be aware of factors that attract nurses with the *right fit* for the program and those that nurses perceive as influencing retention and turnover in the program.

Job embeddedness is a concept arising from organizational, sociology, and psychology literature that is relevant to this analysis. Initially, turnover research emerged with the development of factory labour and introduced job satisfaction and organizational commitment as elements maximizing employee productivity and reducing financial burden for employers [56,57]. As a more recent development, job embeddedness assesses a broad set of influences on employee retention and explains variances beyond those associated with turnover, acknowledging that influences on leaving and staying are not always polar opposites [56]. Mitchell and colleagues [58] recognize three critical aspects of job embeddedness as predictive of voluntary turnover: 1) fit—individuals’ perceptions of their suitability for the position; 2) links—the extent to which employees have connections to other people or activities; and, 3) sacrifice—what employees would give up if they leave the job. Findings from our analysis reinforce, and add to, this narrative.

*Fit*, an aspect of job embeddedness, was reflected in our study findings and associated with recruitment, retention, and turnover. From our analysis, considering fit when recruiting for NFP could help identify nurses that understand public health, home-visitation, and working with families living in complex situations of disadvantage. Recruitment research in healthcare primarily focuses on broad strategies to encourage practitioners into the field to address shortages [11,26,59–61]. Less is known about recruiting for *fit* within nursing. A recent review of value-based recruitment evidence suggested that hiring based on personality, quality, and compassion may be costly upfront, but the benefits may be noted in lower turnover rates [62]. Studies exploring recruitment of new graduate nurses suggest adopting mentorship from more experienced nurses, consistent support, and debriefing opportunities as strategies to encourage entry into positions with positive outcome results and lower turnover [63,64]. Considering these strategies for hiring within NFP may be appropriate given the supports inherently built into the program structure. For example, it may be appropriate to recruit new graduate nurses if they are able to receive guidance from experienced NFP team members and have access to a consistent supervisor. However, we recognize that this may not be possible in organizations where collective bargaining agreements determine seniority over job fit and drive hiring decisions.

Similar to the job embeddedness research [58], our findings suggest that connections made in the NFP team were drivers of retention. Nurses who developed strong connections with their NFP supervisors, team, and clients expressed greater sense of job satisfaction and acknowledged it as a factor related to retention. Another large Canadian study also found that public health nurses are more successful with supportive organizational culture and strong leadership [57]. Conversely, lone NFP nurses, practicing without the immediate face-to-face support of an NFP team, lacked team connection and this consequently influenced nurses’ intent to leave the program. Other research has also found that NFP nurses who work in lone offices experience isolation and recommend strategies to increase connectivity, particularly in rural environments [32]. Decreasing the incidence of lone-office or isolated NFP nurses may help decrease turnover. In rural environments or communities with low client enrollment in NFP, implementing agencies may need to explore opportunities to reduce isolation and increase connection to other NFP team members as described by Campbell et al. [32]. Mechanisms, such as tele-health delivery, are currently being evaluated as measures to reduce isolation and travel costs time for NFP nurses. Future studies could examine the effectiveness of shared-care nursing models and virtual communities of practice in NFP.

Our results indicate that filling NFP positions with public health nurses who identify that their interests, nursing philosophy, and career aspirations align with the work of NFP may facilitate retention. This could be done by posting clearly defined job descriptions and through structured job interviews. In findings similar to ours, Underwood and colleagues [57] suggested that public health nurses prosper with a shared organizational vision, which may increase job satisfaction. Leider et al. [28,29] studied job satisfaction and expected turnover among public health practitioners at local, state, and federal levels and determined that significant turnover can be expected in public health due to factors such as pay and organizational satisfaction. Although remuneration was noted in a previous study from British Columbia, Canada as a factor influencing turnover [10] and mixed findings regarding rate of pay were reported in another Canadian study [57], NFP nurse remuneration did not emerge as an issue in our analysis. This may be because of participants’ structured, unionized environments, as other studies have found nurses working under a collective bargaining agreement have higher satisfaction with their wages [65].

The third aspect of job embeddedness, *sacrifice*, considers what nurses would give up if they leave their positions [58]. Our results suggest that burnout and stress were factors negatively influencing retention. Nurses described sacrificing their own health and wellness if they remained in the NFP. Plendry [66] reported that these symptoms (burnout, stress, etc.) may be indicative of moral distress and can hinder autonomy and reduce nurse retention thus increasing turnover. Recognizing that moral distress is not burnout or stress, we draw on the work of Varcoe et al. [67] and define moral distress as a relational concept, experienced by individuals but shaped by intra and interpersonal factors, as well as broader socio-political and cultural contexts. Nurse autonomy was an important factor in attracting nurses to NFP in our study, therefore, strategies that could facilitate autonomy and reduce moral distress should be explored and encouraged. Moral distress in nursing is “layered and complex”, can be long lasting if not attended to [67, p. 57] and should be addressed as a multi-pronged approach at both organizational and individual levels [68]. More research focused on structural strategies to address moral distress may support nurse retention and reduce turnover in NFP.

For approximately one third of the nurses in our study, leaving NFP was an involuntary or external decision. Our study was situated within the context of a large RCT. This is an important consideration because it affected nurses’ experiences and some organizational decisions ultimately reduced available positions. Although turnover is typically conceptualized as a dysfunctional workforce event [1,8], our findings primarily illuminated what nurses gained by engaging in the NFP program. Even highly experienced public health nurses left the NFP with increased nursing knowledge and skills as a result of their position. This finding suggests that skills may be transferable and could benefit client outcomes in future situations outside NFP. Exiting nurses will be well-situated to inform leadership and policy makers about the NFP program, its benefits and challenges, and advocate for structural changes and resources that support program operation.

We recognize that this analysis is based on the experiences of public health nurses who left their positions after delivering the NFP program in British Columbia. As such, these findings are not intended to be extrapolated to all situations. Instead, we intended to illuminate the experiences of a specific group of nurses who worked in, and then left, the NFP program in a Canadian context. Including nurses who left for voluntary as well as involuntary/external reasons was a strength of this paper. Involuntary and external reasons for turnover are often lacking in turnover research studies. It is a limitation that the process evaluation was conducted within the context of an RCT because this may have influenced nurses’ experiences of delivering NFP independent of their intent to retain or leave the NFP program. This analysis only considered public health nurses’ experiences. Understanding the perspectives of nurses who remained in NFP, supervisors, and senior decision makers may help triangulate findings. As well, understanding motivations for supervisors who decide to leave the NFP program may be an important area for further research.

## Conclusions

The findings from this analysis have significant workforce development implications for public health agencies implementing the NFP program in Canadian settings. They may also inform future broader health human resource planning as health systems shift to population health and primary health care approaches. Public health nurses benefit from a supportive organizational culture and strong nursing leadership. Because NFP program model elements include structured reflection with supervisors and debriefing opportunities with NFP team members, recruiting nurses new to public health may be appropriate. However, attention should be paid to the balance of NFP team experience and all staff should be monitored for factors that can lead to stress and burnout due to the complexity of this type of nursing.

Future research should examine the capacity of NFP supervisors and nurses to prevent and effectively manage moral distress. More research is needed to understand how public health agencies can support public health nurses who work in NFP, and in similar programs, to ensure appropriate support for nurses working with clients dealing with complex health issues. Understanding the nature of unionized environments and their influence on hiring practices in public health nursing could be of interest to NFP implementing agencies as hiring nurses who are a good fit for this work may help prevent turnover. In future analyses, we will explore the influence of delivering NFP in the context of an RCT to determine its impact on nurses’ experiences. Finally, it is important to note that not all turnover is negative and that public health nurses leaving the NFP team take with them an expanded knowledge base and high capacity to provide skilled, complex public health nursing care to other populations experiencing disadvantage.

## Supporting information

S1 File Interview guide. (DOCX)
