# Supplementary material for: “The hardest job you will ever love”: Nurse recruitment, retention, and turnover in the Nurse-Family Partnership program in British Columbia, Canada
Source: PLoS One. 2020 Sep 8;15(9):e0237028. doi: 10.1371/journal.pone.0237028 (PMC7478534; doi:10.1371/journal.pone.0237028)
Supplement: S1 File — (DOCX) [file pone.0237028.s001.docx]

**S1 File.**

**NFP Public Health Nurse Exit Interview Guide**

The questions in this exit interview are divided into two categories and will explore your experiences and perceptions about: 1) Your Position with NFP/BCHCP; and 2) Recommendations for Public Health Nurse Retention.

***In the Nurse-Family Partnership (NFP) model, the public health nurses that deliver the NFP intervention and the Supervisors that support the NFP nurses, are integral parts of NFP and affect the overall effectiveness of the intervention itself. It is also recognized that there are circumstances in which nurses will leave their positions with the NFP/BCHCP for a variety of reasons. It is our hope today that we can learn from you how you came to be involved with the NFP/BCHCP and what circumstances, should you be comfortable sharing these with us, have presented themselves that have resulted in you leaving your position with the NFP/BCHCP.***

*I would like to first ask you how did you learn about the posting for your position/assignment and the opportunity to become involved in the NFP/BCHCP? [Probe for environment in which came into position, whether was unionized, etc.]*

*What motivated you to apply for the position/posting?*

*What were your professional goals at the time that you accepted the position with the NFP/BCHCP?*

*What were your expectations for your role as an NFP public health nurse?*

*How were your goals and expectations a match with your role in delivering the NFP intervention?*

*How were your goals and expectations not a match with your role in delivering the NFP intervention?*

*How did your skills, knowledge, and previous experience, prepare you for your NFP/BCHCP position? [What additional skills and/or knowledge, did they feel were missing at the time of hiring, etc.]*

*What things were provided to you to prepare you for your role delivering the NFP intervention? [Probe for education, reflective supervision, team meetings, program resources etc.]*

*If you were able to speak directly with your replacement, what would you say to them regarding the position that they are coming into? [Probe for most rewarding aspects of position, most challenging aspects of position, what was in place to address these, unanticipated aspects of position that most surprised them, etc.]*

***FOR PART-TIME NFP PUBLIC HEALTH NURSES ONLY***

*In terms of your workload, can you please provide your experience in this role of balancing NFP related work and public health nursing work? [Probe for challenges, successes, loss of non-NFP skillset, work environment, recommendations, etc]*

*If you feel comfortable sharing, what circumstances have presented themselves that have resulted in you leaving your position with NFP/BCHCP? [Probe for job satisfaction, support provided or not from management, another employment opportunity presented, person was covering returning from maternity leave, medical leave, move out of province, relocation due to other circumstances, etc.]*

*Following your work in the NFP program, can you please share with me what your new role and assignment will be? [Probe for whether continuing to provide public health services to similar populations of socially disadvantaged women, description, etc.]*

**One of the overall objectives of today’s interview is to understand from your own perspective as a former NFP nurse your own recommendations for retention of public health nurses moving forward.**

*In reflecting about your experiences working as a public health nurse with the NFP/BCHCP, what strategies currently exist that you are aware of, if any, to contribute to retention of nurses? [Probe for if any of these strategies were utilized to retain participant]*

*In reflecting about your experiences working as a public health nurse with the NFP/BCHCP, what additional strategies would you recommend be integrated into the BCHCP in order to contribute to increased retention of public health nurses? [Probe for whether including these new strategies might have affected participants’ own retention in the BCHCP]*

*What factors should be considered when recruiting new NFP nurses? [Probe for factors related to skillset, training, and education]*

As we wrap our conversation today, are there any additional comments that you would like to share with the research team about:

a) Your Position with NFP/BCHCP;

b) Recommendations for public health nurses;

c) Other Comments.
